# Museomics allows comparative analyses of mitochondrial genomes in the family Gryllidae (Insecta, Orthoptera) and confirms its phylogenetic relationships

**DOI:** 10.7717/peerj.17734

**Published:** 2024-08-08

**Authors:** Jiajia Dong, Yong Liu, Ming Kai Tan, Rodzay Abdul Wahab, Romain Nattier, Pascaline Chifflet-Belle, Tony Robillard

**Affiliations:** 1 School of Life Sciences, Jiangsu Key Laboratory of Brain Disease and Bioinformation, Research Center for Biochemistry and Molecular Biology, Xuzhou Medical University, Xuzhou, China; 2 Institut de Systématique, Evolution et Biodiversité (ISYEB), Muséum national d’Histoire naturelle, CNRS, SU, EPHE-SPL, UA, Paris, France; 3 Institute for Biodiversity and Environmental Research, Universiti Brunei Darussalam, Jalan Universiti, Gadong, Brunei Darussalam

**Keywords:** Biological collection, NGS, Eneopterinae, Mitogenome, Molecular phylogeny, Cricket, Taxonomy

## Abstract

**Background:**

Next-generation sequencing technology can now be used to sequence historical specimens from natural history collections, an approach referred to as museomics. The museomics allows obtaining molecular data from old museum-preserved specimens, a resource of biomolecules largely underexploited despite the fact that these specimens are often unique samples of nomenclatural types that can be crucial for resolving scientific questions. Despite recent technical progress, cricket mitogenomes are still scarce in the databases, with only a handful of new ones generated each year from freshly collected material.

**Methods:**

In this study, we used the genome skimming method to sequence and assemble three new complete mitogenomes representing two tribes of the cricket subfamily Eneopterinae: two were obtained from old, historical type material of *Xenogryllus lamottei* (68 years old) and *X. maniema* (80 years old), the third one from a freshly collected specimen of *Nisitrus vittatus*. We compared their genome organization and base composition, and reconstructed the molecular phylogeny of the family Gryllidae.

**Results:**

Our study not only confirmed that the genome skimming method used by next generation sequencing allows us to efficiently obtain the whole mitogenome from dry-pinned historical specimens, but we also confirmed how promising it is for large-scale comparative studies of mitogenomes using resources from natural history collections. Used in a phylogenetic context the new mitogenomes attest that the mitogenomic data contain valuable information and also strongly support phylogenetic relationships at multiple time scales.

## Introduction

True crickets (superfamily Grylloidea Laicharting, 1781) is one of the most diverse lineages in Orthoptera (Song et al., 2015) and is well known for acoustic signals produced in the contexts of courtship and mate recognition (Desutter, 1988; Gerhardt & Huber, 2002). Based on recent phylogenetic studies (Chintauan-Marquier et al., 2016; de Campos et al., 2022), five extant families corresponding to monophyletic clades are currently recognized in the superfamily Grylloidea: Gryllidae Laicharting, 1781, Mogoplistidae Costa, 1855, Oecanthidae Blanchard, 1845, Phalangopsidae Blanchard, 1845, and Trigonidiidae Saussure, 1874. Within the family Gryllidae, the subfamily Eneopterinae Saussure, 1874 comprises five monophyletic tribes (Tan et al., 2021). Eneopterinae is a diversified clade distributed around the world in most tropical areas (*e.g.*, Robillard & Desutter-Grandcolas, 2004; Vicente et al., 2017) and characterized by diverse shapes and original features related to acoustic communication, including complex acoustic behaviors and structures (Desutter-Grandcolas, 1998; Robillard & Desutter-Grandcolas, 2006; Robillard & Desutter-Grandcolas, 2011; Tan et al., 2021), the use of high-frequency songs in males and vibrational response in females (*e.g.*, Benavides-Lopez, Hofstede & Robillard, 2020; Robillard & Desutter-Grandcolas, 2004; Robillard et al., 2013; Robillard et al., 2015; ter Hofstede et al., 2015). Recent studies investigated their diversity through phylogenetic and biogeographical studies (Dong et al., 2018; Nattier et al., 2011; Vicente et al., 2017), but the lack of historical material resulted in specific key taxa being excluded from the otherwise well-sampled data. To better understand the origin and evolution of the unique features of these species, we need a robust phylogenetic framework in the context of the phylogenetic reconstruction of the family Gryllidae. In comparison to single/multi-locus mitochondrial DNA (mtDNA) markers, the use of complete mitochondrial genomic data (mitogenome) is a good compromise to sampling both a large amount of data and a large number of species, including some with very few specimens available, to reconstruct the phylogenetic backbone of the group under study (*e.g.*, Nie et al., 2020).

The insect mitogenome is a compact circular molecule (15–18 k bp) encoding a conserved set of 13 protein-coding genes (PCGs), 22 tRNA genes, and two rRNA genes. Mitogenome is a convenient option for phylogenetic reconstruction for several reasons: (1) due to the high number of copies of this molecule, it is relatively easy to obtain mitochondrial molecular markers; (2) although it is variable enough to be used for barcoding initiatives, the mitochondrial genes are characterized by diverse rates of evolution, which make them informative markers for phylogenetic reconstruction at multiple taxonomic scales (Crampton-Platt et al., 2015; Crampton-Platt et al., 2016; Timmermans et al., 2010); (3) while rare exceptions of rearrangement have been found, the arrangements of insect mitogenomes are relatively stable, which promise mitogenomes as a useful data set for the study of deep divergences of insects (Cameron, 2014). On the other hand, the rearrangements in some lineages indicate diversities within the group, such as D-K (D: tRNA-Asp or *trnD*, K: tRNA-Lys or *trnK*) rearrangement in the lineage Acridomorpha (Li et al., 2020) and some crickets, inversion of the gene cluster M-I-Q (M: tRNA-Met or *trnM*, I: tRNA-Ile or *trnI*, Q: tRNA-Gln or *trnQ*) and occasional inversion of W (tRNA-Trp or *trnW*) (Ma et al., 2019). Moreover, considering the potential effect of nuclear copies of mitochondrial genes, or pseudogenes (numts), the complete mitogenomes in crickets contain more accurate phylogenetic information than single or multi-locus data (Song et al., 2008). Mitogenomes have consequently been considered as a powerful source of data (combing appropriate models) to reconstruct phylogenies accuracy, power and robustness with strong branch supports (Nie et al., 2020; Song et al., 2020).

With the development of the Next Generation Sequencing (NGS) technologies, obtaining the mitogenomic information from fresh or ethanol-preserved specimens is now easy. However, the amplification of mitogenomes from historical specimens remains more challenging because of DNA degradation and fragmentation, even when obtaining some short fragments is also becoming common (*e.g.*, *cox1* barcoding in insects (Strutzenberger, Brehm & Fiedler, 2012)). Genome skimming, a shallow NGS approach that allows for comparatively deep sequencing of high-copy genomes such as the mitogenome and complete nuclear ribosomal cluster (Dodsworth, 2015; Straub et al., 2012), has been used successfully at varying taxonomic levels, *e.g.*, in octopus (Taite et al., 2023), plants (Liu et al., 2021), and even pathogens (Denver et al., 2016). Despite the technical progress and the current trend of using complete mitogenomic data for phylogenetic analyses in insects, including in ensiferans (Song et al., 2020; Zhou et al., 2017), cricket mitogenomes are still very scarce in the data bases, with only a handful of new ones generated each year from on freshly collected material.

In this study, we sequenced and assembled three new complete mitogenomes, representing two tribes of subfamily Eneopterinae by using the genome skimming method. Among these, two were obtained from old historical type material, while one corresponds to a freshly collected specimen, which allows direct comparisons of the efficiency of the methods used to recover mitogenomic data of the superfamily Grylloidea.

## Materials and methods

### Sampling and genomic DNA extraction

New sequence data correspond to three species of the cricket subfamily Eneopterinae. One specimen of the species *Nisitrus vitattus* (Haan, 1844) (tribe Nisitrini) was collected recently in Brunei, preserved in absolute ethyl alcohol and deposited in Muséum national d’Histoire naturelle, Paris (MNHN). The collection of *N. vittatus* material in Kuala Belalong Field Studies Centre, Brunei Darussalam was granted by the Institute for Biodiversity and Environmental Research, Universiti Brunei Darussalam (UBD/AVC-RI/1.21.1 [a]) and the export permit was issued by the Research Innovation Centre (BioRIC), Ministry of Primary Resources and Tourism, Brunei Darussalam (BioRIC/HoB/TAD/51-73). The two other specimens correspond to historical dry insect specimens from MNHN and Musée Royal de l’Afrique Centrale, Tervuren, Belgium (MRAC), which were selected during the recent taxonomic revision of the genus *Xenogryllus* Bolívar, 1890 (Jaiswara et al., 2019): the holotype and unique specimen of *Xenogryllus lamottei* Robillard, 2019 collected in Guinea (Simandou Mount) in 1951, and one male paratype of *Xenogryllus maniema* Robillard & Jaiswara, 2019 collected in 1939. The sampling information in detail is presented in Table S1.

DNA extraction, PCR amplification, and bank preparations were carried out at Service de Systématique Moléculaire of the MNHN (SSM). Total genomic DNA of the three species were extracted from a median leg, using a DNeasy Blood and Tissue Kit (Qiagen Inc., Valencia, CA, USA) following the manufacturer’s instructions. We used this method as it is a non-destructive way to prevent damaging legs of type specimens, which were dried after DNA extraction and replaced on the specimens. The three extracts were then used for library preparation in a genome skimming approach (Straub et al., 2012) as presented in Salazar et al. (2020) with a minor modification: the recent specimen *N. vitattus* (molecular code: N37) underwent DNA fragmentation in sonication step, while the older specimens *X. lamottei* (molecular code: X24) and *X. maniema* (molecular code: X36) skipped that step due to their age and a more fragmented DNA.

### Mitochondrial genome sequencing and assembly

The workflow including sequencing reads quality detection from both paired-end libraries, the interest sequences extraction from the total reads and the *de novo assemble* procedure was followed the protocol of a previous study (Salazar et al., 2020). The mitochondrial genomes of *Xenogryllus marmoratus* (Haan, 1844) (Ma, Zhang & Li, 2019): Genbank accession MK033622) was used as reference in the *Map to reference* option in Geneious.

### Mitochondrial genome annotation and sequence analyses

Annotation of the mitogenomes was performed using the MITOS webserver with invertebrate genetic code (Bernt et al., 2013) and modified after comparisons with other mitogenomes from Grylloidea species. The validation of tRNA sequences was performed in tRNAscan-SE (http://trna.ucsc.edu/tRNAscan-SE/) and ARWEN (http://130.235.244.92/ARWEN/) using the invertebrate mitogenome genetic codon (Chan et al., 2019; Laslett & Canback, 2008). The tandem repeats in the control regions were found with Tandem Repeats Finder web server (TRF version 4.09, (Benson, 1999)).

The nucleotide base compositions of the complete mitogenomes were calculated in Geneious 9.0.2 (Biomatter Ltd., New Zealand, http://www.geneious.com, Kearse et al., 2012). The nucleotide compositional skews were calculated following the formula by Konstantinov et al. (2016): AT-skew = (A-T)/(A+T) and GC-skew = (G-C)/(G+C), where A, C, G, and T are the frequencies of the four bases. The nucleotide base composition comparison analysis for the whole mitogenomes, two ribosomal genes (the large and small ribosomal subunit, *rrnL*, and *rrnS*) among 32 Gryllidae mitogenomes (29 sequences from GenBank and three news in this study) were also calculated. The PCGs nucleotide composition, genome and codon position were determined and the PCGs were translated into a sequences of amino acid residues *in silico* for calculating of the relative synonymous codon usage (RSCU) using the invertebrate mitochondrial genetic code in MEGA 7.0 (Kumar, Stecher & Tamura, 2016). Besides, to detect the selective pressure of eneopterine mitogenomes, the comparison analyses of synonymous and non-synonymous substitutions (Ka/Ks) for each PCG in eneopterine crickets were calculated using KaKs_Calculator 3.0 software (https://ngdc.cncb.ac.cn/biocode/tools/BT000001) (Zhang, 2022).

### Phylogenetic analysis

Thirteen protein-coding genes, 22 tRNAs and 2 rRNAs of 32 Gryllidae species (29 from GenBank and three in this study), and six species representing other families in the superfamily Grylloidea were used for the phylogenetic analysis. Four species representing the Gryllotalpoidea superfamily were used as outgroup. The detailed information about the taxonomic sampling used in this study is listed in Table S2.

The phylogenetic analysis was applied in Bayesian Inference and Maximum Likelihood using mitogenomes in PhyloSuite V1.2.3 program (http://phylosuite.jushengwu.com/dongzhang0725.github.io/installation/) following the users’ instructions (Xiang et al., 2023; Zhang et al., 2020). For BI tree, a clade with a PP value higher than 0.95 was considered as strongly supported following Erixon et al. (2003). For ML tree, nodes supported by bootstrap support values (BS) ≥70% were considered strongly supported following Hillis & Bull (1993).

## Results

### Sequencing and assembly of mitochondrial genome

After excluding low quality reads from the sequencing, a total of 15,765,390 read-pairs were generated from the recent specimen of *Nisitrus vittatus* (collected in 2017); 989,605 read-pairs from the holotype of *Xenogryllus lamottei* collected in 1951; and 2,127,394 read-pairs from the paratype of *Xenogryllus maniema* collected in 1939.

After a “Map to Reference” assembly in Geneious R9.0.2, 56,224 reads were assembled to the *X. lamottei* bait sequence, 33,572 reads to the *X. maniema* bait sequence, and 31,196 reads to the *N. vittatus* bait sequence. For the quality examination of the contigs, pairwise identity reached 99.9% in the three cricket species. Consensus assemblies were generated, which were 16,156 bp long for *X. lamottei*, 16,191 bp for *X. maniema* and 15,458 bp for *N. vittatus*. Overlapped regions were manually removed to circularize the complete mitochondrial genome of *X. lamottei*, *X. maniema*, and *N. vittatus* (15,590 bp, 15,853 bp, and 15,450 bp, respectively). The three complete mitochondrial genome sequences were deposited in GenBank under accession numbers OQ459859 (*N. vittatus*, molecular code: N37), OQ457268 (*X. lamottei*, molecular code: X24), and OQ457269 (*X. maniema*, molecular code: X36).

### Genome organization and base composition

**Figure 1:**
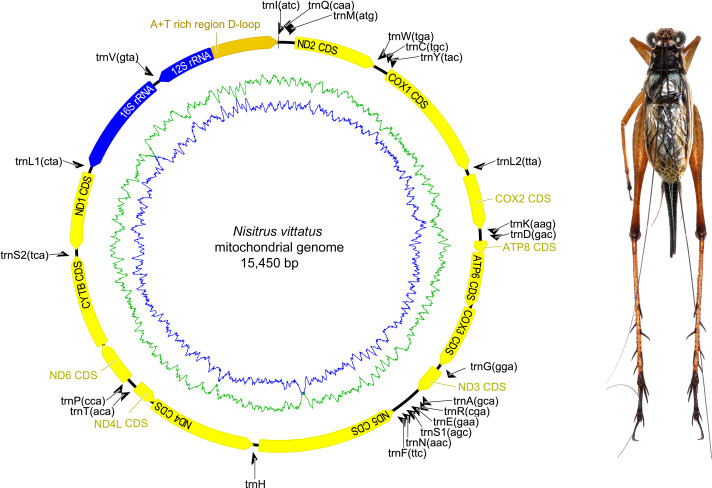
Mitochondrial genome organizations of *Nisitrus vittatus*. Circular maps on the left indicates mitochondrial genome organizations, and the corresponding species in dorsal view were showed on the right. Circular maps exported by Geneious R9.0.2 and photo of male specimen in dorsal view were taken by MKT using the photography platform of the MNHN collections. The figure was assembled using Adobe Illustrator 24.1. The orientation of gene transcription is indicated with arrows. The tRNA genes are indicated with their one-letter corresponding amino acids. The GC content and AT content were plotted using a blue and green sliding window in the circular map respectively, as the deviation from the average GC content and AT content of the entire sequence. Circular map was drawn using Geneious 9.0.2.

**Figure 2:**
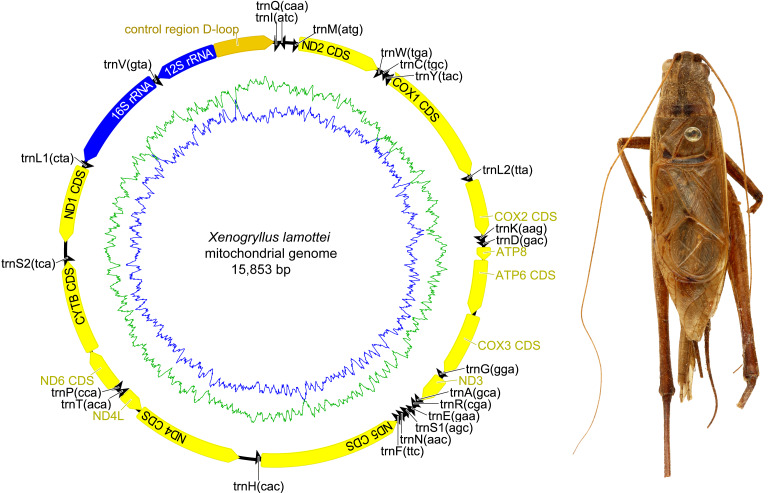
Mitochondrial genome organizations of *Xenogryllus lamottei*. Circular maps exported by Geneious R9. 0.2 and photo of male holotype in dorsal view were taken by TR using the photography platform of the MNHN collections. Circular map was drawn using Geneious 9.0.2.

**Figure 3:**
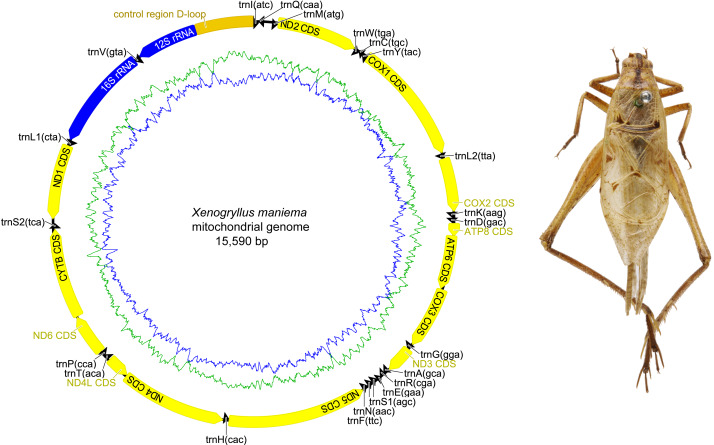
Mitochondrial genome organizations of *Xenogryllus maniema*. Circular maps exported by Geneious R9. 0.2 and photo of males paratype specimen in dorsal view were taken by TR using the photography platform of the MNHN collections. Circular map was drawn using Geneious 9.0.2.

The complete mitochondrial genome organization of the three eneopterine crickets was consistent with the ancestral insect mitochondrial genome (Cameron, 2014), including 13 PCGs, two rRNA genes, 22 tRNA genes (one for each amino acid, two for Leucine and Serine), and a major non-coding region known as the control region, CR (Figs. 1–3 & Table 1). Twenty genes were transcribed on the majority strand (J-strand) and the other 17 genes were oriented on the minority strand (N-strand). Moreover, the ancestral gene arrangement of a local inversion of the *trnN*-*trnS1*-*trnE* to *trnE*-*trnS1*-*trnN* were observed in the three eneopterine crickets, which is consistent with the published gryllid mitogenomes (Yang, Ren & Huang, 2016; Zhou et al., 2017). For the three species, the overlapping sequences of all ranged in size from 1 to 10 bp, while there was a discrepancy in the length of the intergenic spacers between *N. vittatus* and the two *Xenogryllus* species. The intergenic spacer sequences range from 1 to 189 bp in *X. lamottei* and 1 to 103 bp in *X. maniema* (Table 1). In *X. lamottei*, the three longest intergenic spacers were located between *trnQ* and *trnM* (94 bp), between *trnS2* and *nd1* (155 bp), and between *trnH* and *nd4* (189 bp). In *X. maniema*, they were located between *nd4l* and *trnT* (12 bp), *trnS2* and *nd1* (47 bp), and between *trnQ* and *trnM* (103 bp). However, the longest intergenic spacers in *N. vittatus* were 21 bp (located between *nd1* and *trnS2*), 7 bp (located between *atp6* and *atp8*), and 4 bp (located between *nd4l* and *trnT*).

**Table 1:** List of annotated mitochondrial genes of *Xenogryllus lamottei*, *Xenogryllus maniema* and *Nisitrus vittatus*. The protein coding and ribosomal RNA genes are represented by standard nomenclature, tRNAs are represented as *trn* followed by the IUPAC-IUB single letter amino acid codes. (J) values in strand represent as majority strand (J-strand) and (N) values represent as minority strand (N-strand). IGS represents (+) values as intergenic spacer and (−) values as overlapping regions. CR represents the control region.

	*Xenogryllus lamottei*					*Xenogryllus maniema*					*Nisitrus vittatus*				
	Strand	Location	Size (bp)	Start/stop codon	IGS	Strand	Location	Size (bp)	Start/stop codon	IGS	Strand	Location	Size (bp)	Start/stop codon	IGS
*trnI*	J	1–65	65			J	1–63	63			J	1–64	64		
*trnQ*	N	63–131	69		−3	N	60–130	71		−4	N	61–131	71		−4
*trnM*	J	226–294	69		94	J	234–302	69		103	J	131–199	69		−1
*nd2*	J	295–1308	1,014	ATT/TAG	0	J	303–1316	1,014	ATT/TAA	0	J	200–1,219	1,020	ATT/TAA	0
*trnW*	J	1,309–1,371	63		0	J	1,313–1,381	69		−4	J	1,217–1,284	68		−3
*trnC*	N	1,364–1,425	62		−8	N	1,372–1,433	62		−10	N	1,276–1,338	63		−9
*trnY*	N	1,431–1,497	67		5	N	1,436–1,502	67		2	N	1,342–1,408	67		3
*cox1*	J	1,496–3,032	1,537	TCG/T	−2	J	1,495–3,037	1,543	ATC/T	−8	J	1,401–2,945	1,545	ATT/TAA	−8
*trnL2*	J	3,032–3,100	69		−1	J	3,038–3,104	67		0	J	2,940–3,005	66		−6
*cox2*	J	3,103–3,778	676	ATG/T	2	J	3,105–3,780	676	ATG/T	0	J	3,008-3,685	678	ATG/TAA	2
*trnK*	J	3,778–3,849	72		−1	J	3,780–3,851	72		−1	J	3,688–3,758	71		2
*trnD*	J	3,848–3,913	66		−2	J	3,850–3,916	67		−2	J	3,758–3,823	66		−1
*atp8*	J	3,914–4,075	162	ATT/TAA	0	J	3,917–4,081	165	ATT/TAA	0	J	3,824–3,979	156	ATC/TAA	0
*atp6*	J	4,069–4,752	684	ATG/TAA	−7	J	4,075–4,758	684	ATG/TAA	−7	J	3,973–4,656	684	ATG/TAA	7
*cox3*	J	4,752–5,540	789	ATG/TAA	−1	J	4,758–5,544	787	ATG/T	−1	J	4,656–5,442	787	ATG/T	−1
*trnG*	J	5,543–5,607	65		2	J	5,544–5,609	66		0	J	5,442–5,506	65		−1
*nd3*	J	5,608–5,961	354	ATC/TAA	0	J	5,609–5,962	354	ATA/TAA	−1	J	5,506–5,859	354	ATC/TAG	−1
*trnA*	J	5,963–6,028	66		1	J	5,964–6,029	66		1	J	5,857–5,923	67		−3
*trnR*	J	6,029–6,090	62		0	J	6,030–6,091	62		0	J	5,922–5,984	63		−2
*trnE*	N	6,084–6,150	67		−7	N	6,086–6,150	65		−6	N	5,978–6,041	64		−7
*trnS1*	N	6,152–6,218	67		1	N	6,153–6,223	71		2	N	6,043–6,109	67		1
*trnN*	N	6,219–6,284	66		0	N	6,221–6,289	69		−3	N	6,110–6,178	69		0
*trnF*	N	6,284–6,348	65		−1	N	6,298–6,362	65		8	N	6,179–6,241	63		0
*nd5*	N	6,348–8,094	1,747	ATT/T	−1	N	6,362–8,105	1,744	ATT/T	−1	N	6,243–7,968	1,726	ATT/T	1
*trnH*	N	8,095–8,160	66		0	N	8,105–8,171	67		−1	N	7,969–8,030	62		0
*nd4*	N	8,350–9,691	1,342	ATG/T	189	N	8,171–9,512	1,342	ATG/T	−1	N	8,031–9,366	1,336	ATG/T	0
*nd4l*	N	9,685–9,981	297	ATG/TAA	−7	N	9,506–9,802	297	ATG/TAA	−7	N	9,360–9,656	297	ATG/TAA	−7
*trnT*	J	9,987–10,053	67		5	J	9,815–9,878	64		12	J	9,661–9,728	68		4
*trnP*	N	10,051–10,117	65		−3	N	9,879–9,943	65		0	N	9,726–9,792	67		−3
*nd6*	J	10,119–10,640	522	ATT/TAA	1	J	9,946–10,470	525	ATA/TAA	2	J	9,794–10,315	522	ATT/TAA	1
*cytb*	J	10,640–11,776	1,137	ATG/TAA	−1	J	10,470–11,606	1,137	ATG/TAA	−1	J	10,319–11,455	1,137	ATG/TAG	3
*trnS2*	J	11,778–11,844	67		1	J	11,614–11,680	67		7	J	11,454–11,517	64		−2
*nd1*	N	12,000–12,938	939	TTG/TAG	155	N	11,728–12,669	942	TTG/TAG	47	N	11,539–12,478	940	TTG/T	21
*trnL1*	N	12,938–13,007	70		−1	N	12,669–12,738	70		−1	N	12,478–12,543	66		−1
*rrnL*	N	13,007–14,293	1,286		−1	N	12,738–14,025	1,288		0	N	12,543–13,816	1,274		−1
*trnV*	N	14,294–14,361	68		0	N	14,026–14,093	68		0	N	13,817–13,886	70		0
*rrnS*	N	14,361–15,132	772		−1	N	14,093–14,868	776		−1	N	13,890–14,637	748		3
*CR*	J	15,133–15,853	721		0	J	14,869–15,590	722		0	J	14,638–15,450	813		0

**Figure 4:**
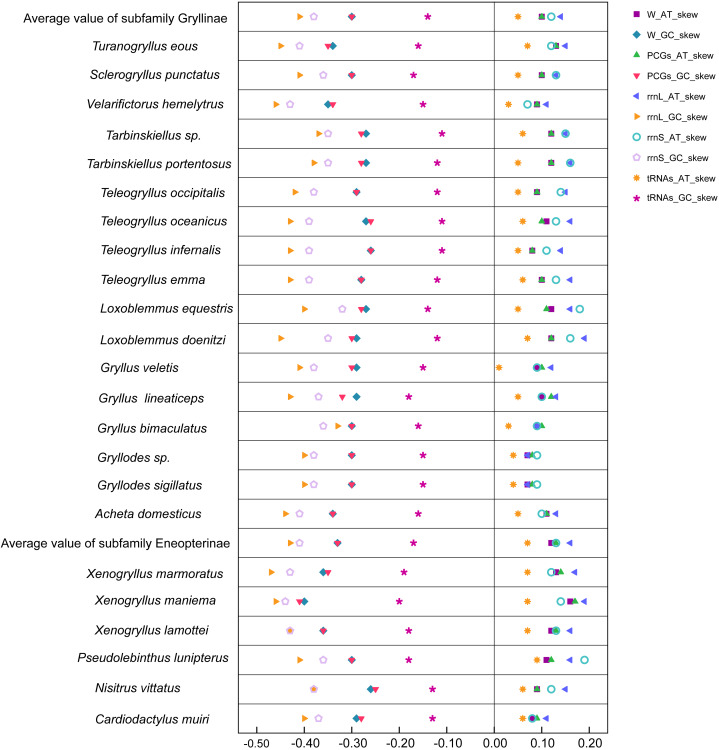
Comparison of AT-skews and GC-skews of Gryllidae family. Legends on the right-top indicated AT skew and GC skew (ending with suffix as AT_skew and GC_skew) for whole mitochondrial genomes, protein coding genes, two ribosomal RNA genes and transfer RNA genes, starting with prefix as W_, PCGs_, rrnL_, rrnS_, and tRNAs_, separately.

The nucleotide composition of three mitogenomes were typically insect-A+T biased (with 74.8% in *N. vittatus*, 72.9% in *X. lamottei*, and 69.6% in *X. maniema*, respectively), and were slightly A skewed (AT-skew = 0.09 in *N. vittatus*, 0.12 in *X. lamottei*, and 0.16 in *X. maniema*, respectively) and strongly C skewed (GC-skew = −0.26 in *N. vittatus*, −0.36 in *X. lamottei*, and −0.40 in *X. maniema*, respectively). The nucleotide compositional skew of the whole mitogenomes, PCGs genes, *rrnL* genes, *rrnS* genes, and tRNAs genes of Gryllidae crickets were also represented in Table 2 and Fig. 4. The nucleotide composition of the control region were not represented in Fig. 4 (but partially shown in Table 2) as a result of unconfirmed by the original data from the GenBank.

**Table 2:** General nucleotide composition of complete mitogenome and nucleotide compositional skew of the Gryllidae mitogenomes, rrnL genes, rrnS genes, and the control region in grylline crickets. A (%), T (%), G (%), and C (%) mean the percentage of adenine, thymine, guanine, and cytosine. The number with right-top letters represent the average nucleotide compositional skews in the subfamilies Eneopterinae (a) and Gryllinae (b). The numbers with left-top asterisks ‘*’ represent the average nucleotide compositional skews for this species. Signs ‘-’ indicate nucleotide compositional skews not calculated.

	Whole mitogenome						
Species	Length (bp)	A (%)	T (%)	G (%)	C (%)	AT_skew	GC_skew
*Cardiodactylus muiri*	16,328	41.4	35.1	8.4	15.2	0.08	−0.29
*Nisitrus vittatus*	15,450	40.9	33.9	9.3	15.8	0.09	−0.26
*Pseudolebinthus lunipterus*	16,075	42.1	33.7	8.5	15.7	0.11	−0.30
*Xenogryllus lamottei*	15,853	40.8	32.1	8.7	18.5	0.12	−0.36
*Xenogryllus maniema*	15,590	40.2	29.4	9.1	21.3	0.16	−0.40
*Xenogryllus marmoratus*	15,762	40.8	31.3	8.7	19.2	0.13	−0.38
*Xenogryllus marmoratus*	15,576	40.5	31.3	9.3	19	0.13	−0.34
*Xenogryllus marmoratus						0.13	−0.36
subfamily Eneopterinae						0.12^a^	−0.33^a^
*Acheta domesticus*	15,784	39.2	31.7	9.5	19.6	0.11	−0.35
*Acheta domesticus*	16,071	39.5	31.9	9.4	19.1	0.11	−0.34
*Acheta domesticus						0.11	−0.34
*Gryllodes sigillatus*	16,369	37.8	32.6	10.3	19.3	0.07	−0.30
*Gryllodes sigillatus*	16,176	37.9	32.9	10.1	19	0.07	−0.31
*Gryllodes sigillatus						0.07	−0.30
*Gryllodes* sp.	15,550	37.9	32.7	10.2	19.1	0.07	−0.30
*Gryllus bimaculatus*	16,075	40.3	33.8	9.1	16.8	0.09	−0.30
*Gryllus bimaculatus*	15,737	40.5	33.7	9	16.8	0.09	−0.30
*Gryllus bimaculatus						0.09	−0.30
*Gryllus lineaticeps*	15,607	39.9	32.7	9.7	17.8	0.10	−0.29
*Gryllus veletis*	15,686	40.1	33.5	9.4	16.9	0.09	−0.29
*Loxoblemmus doenitzi*	15,396	40.9	32.4	9.4	17.3	0.12	−0.30
*Loxoblemmus doenitzi*	15,399	40.9	32.2	9.5	17.3	0.12	−0.29
*Loxoblemmus doenitzi						0.12	−0.29
*Loxoblemmus equestris*	16,314	40.3	31.6	10.2	17.9	0.12	−0.27
*Teleogryllus emma*	15,697	40.2	33	9.6	17.2	0.10	−0.28
*Teleogryllus emma*	16,044	40.4	33.2	9.5	17	0.10	−0.28
*Teleogryllus emma*	15,660	40.5	32.6	9.8	17	0.11	−0.27
*Teleogryllus emma						0.10	−0.28
*Teleogryllus infernalis*	15,512	40	33.9	9.6	16.5	0.08	−0.26
*Teleogryllus oceanicus*	15,660	40.4	32.6	9.8	17.1	0.11	−0.27
*Teleogryllus occipitalis*	15,501	40.2	33.3	9.5	17.1	0.09	−0.29
*Teleogryllus occipitalis*	15,501	40.2	33.3	9.5	17.1	0.09	−0.29
*Teleogryllus occipitalis						0.09	−0.29
*Tarbinskiellus portentosus*	15,710	40.6	32.2	9.9	17.3	0.12	−0.27
*Tarbinskiellus portentosus*	15,498	40.6	32.2	9.9	17.3	0.12	−0.27
*Tarbinskiellus portentosus						0.12	−0.27
*Tarbinskiellus* sp.	15,514	40.6	32.2	9.9	17.3	0.12	−0.27
*Velarifictorus hemelytrus*	16,123	39.7	32.9	8.9	18.4	0.09	−0.35
*Sclerogryllus punctatus*	15,438	41	33.7	8.8	16.4	0.10	−0.30
*Turanogryllus eous*	16,045	40.1	31	9.6	19.4	0.13	−0.34
subfamily Gryllinae						0.10^b^	−0.30^b^

	PCGs		rrnLs		rrnSs	
Species	AT_skew	GC_skew	AT_skew	GC_skew	AT_skew	GC_skew
*Cardiodactylus muiri*	0.09	−0.28	0.11	−0.40	0.08	−0.37
*Nisitrus vittatus*	0.09	−0.25	0.15	−0.38	0.12	−0.38
*Pseudolebinthus lunipterus*	0.12	−0.30	0.16	−0.41	0.19	−0.36
*Xenogryllus lamottei*	0.13	−0.36	0.16	−0.43	0.13	−0.43
*Xenogryllus maniema*	0.17	−0.41	0.19	−0.46	0.14	−0.44
*Xenogryllus marmoratus*	0.15	−0.37	0.17	−0.47	0.12	−0.44
*Xenogryllus marmoratus*	0.14	−0.34	0.17	−0.46	0.12	−0.43
*Xenogryllus marmoratus	0.14	−0.35	0.17	−0.47	0.12	−0.43
subfamily Eneopterinae	0.13^a^	−0.33^a^	0.16^a^	−0.43^a^	0.13^a^	−0.41^a^
*Acheta domesticus*	0.11	−0.34	0.13	−0.44	0.10	−0.41
*Acheta domesticus*	0.11	−0.34	0.12	−0.44	0.10	−0.41
*Acheta domesticus	0.11	−0.34	0.13	−0.44	0.10	−0.41
*Gryllodes sigillatus*	0.08	−0.30	0.07	−0.40	0.09	−0.38
*Gryllodes sigillatus*	0.08	−0.30	0.07	−0.41	0.09	−0.38
*Gryllodes sigillatus	0.08	−0.30	0.07	−0.40	0.09	−0.38
*Gryllodes* sp.	0.08	−0.30	0.07	−0.40	0.09	−0.38
*Gryllus bimaculatus*	0.10	−0.30	0.07	−0.30	0.09	−0.34
*Gryllus bimaculatus*	0.10	−0.30	0.12	−0.37	0.10	−0.38
*Gryllus bimaculatus	0.10	−0.30	0.09	−0.33	0.09	−0.36
*Gryllus lineaticeps*	0.12	−0.32	0.13	−0.43	0.10	−0.37
*Gryllus veletis*	0.10	−0.30	0.12	−0.41	0.09	−0.38
*Loxoblemmus doenitzi*	0.12	−0.31	0.19	−0.45	0.16	−0.35
*Loxoblemmus doenitzi*	0.12	−0.30	0.19	−0.44	0.16	−0.35
*Loxoblemmus doenitzi	0.12	−0.30	0.19	−0.45	0.16	−0.35
*Loxoblemmus equestris*	0.11	−0.28	0.16	−0.40	0.18	−0.32
*Teleogryllus emma*	0.09	−0.28	0.16	−0.41	0.14	−0.39
*Teleogryllus emma*	0.09	−0.29	0.16	−0.43	0.14	−0.38
*Teleogryllus emma*	0.10	−0.26	0.16	−0.44	0.13	−0.39
*Teleogryllus emma	0.10	−0.28	0.16	−0.43	0.13	−0.39
*Teleogryllus infernalis*	0.08	−0.26	0.14	−0.43	0.11	−0.39
*Teleogryllus oceanicus*	0.10	−0.26	0.16	−0.43	0.13	−0.39
*Teleogryllus occipitalis*	0.09	−0.29	0.15	−0.43	0.14	−0.38
*Teleogryllus occipitalis*	0.09	−0.28	0.15	−0.42	0.14	−0.38
*Teleogryllus occipitalis	0.09	−0.29	0.15	−0.42	0.14	−0.38
*Tarbinskiellus portentosus*	0.12	−0.28	0.15	−0.38	0.15	−0.36
*Tarbinskiellus portentosus*	0.12	−0.28	0.16	−0.38	0.16	−0.35
*Tarbinskiellus portentosus	0.12	−0.28	0.16	−0.38	0.16	−0.35
*Tarbinskiellus* sp.	0.12	−0.28	0.15	−0.37	0.15	−0.35
*Velarifictorus hemelytrus*	0.09	−0.34	0.11	−0.46	0.07	−0.43
*Sclerogryllus punctatus*	0.10	−0.30	0.13	−0.41	0.13	−0.36
*Turanogryllus eous*	0.13	−0.35	0.15	−0.45	0.12	−0.41
subfamily Gryllinae	0.10^b^	−0.30^b^	0.14^b^	−0.41^b^	0.12^b^	−0.38^b^

	tRNAs		Control regions	
Species	AT_skew	GC_skew	AT_skew	GC_skew
*Cardiodactylus muiri*	0.06	−0.13	0.01	−0.37
*Nisitrus vittatus*	0.06	−0.13	0.07	−0.24
*Pseudolebinthus lunipterus*	0.09	−0.18	−0.06	−0.28
*Xenogryllus lamottei*	0.07	−0.18	0.02	−0.35
*Xenogryllus maniema*	0.07	−0.20	0.04	−0.45
*Xenogryllus marmoratus*	0.07	−0.19	0.00	−0.39
*Xenogryllus marmoratus*	0.08	−0.18	−0.03	−0.28
*Xenogryllus marmoratus	0.07	−0.19		
subfamily Eneopterinae	0.07^a^	−0.17^a^		
*Acheta domesticus*	0.05	−0.16	–	–
*Acheta domesticus*	0.05	−0.16	–	–
*Acheta domesticus	0.05	−0.16		
*Gryllodes sigillatus*	0.04	−0.15	0.09	−0.32
*Gryllodes sigillatus*	0.04	−0.15	0.05	−0.31
*Gryllodes sigillatus	0.04	−0.15		
*Gryllodes* sp.	0.04	−0.15	–	–
*Gryllus bimaculatus*	0.03	−0.15	–	–
*Gryllus bimaculatus*	0.03	−0.16	–	–
*Gryllus bimaculatus	0.03	−0.16		
*Gryllus lineaticeps*	0.05	−0.18	−0.11	0.05
*Gryllus veletis*	0.01	−0.15	0.01	−0.09
*Loxoblemmus doenitzi*	0.07	−0.12	0.01	−0.03
*Loxoblemmus doenitzi*	0.07	−0.12	0.02	−0.04
*Loxoblemmus doenitzi	0.07	−0.12		
*Loxoblemmus equestris*	0.05	−0.14	0.22	−0.20
*Teleogryllus emma*	0.06	−0.13	0.13	−0.18
*Teleogryllus emma*	0.05	−0.12	–	–
*Teleogryllus emma*	0.06	−0.10	0.13	−0.23
*Teleogryllus emma	0.06	−0.12		
*Teleogryllus infernalis*	0.05	−0.11	0.10	−0.11
*Teleogryllus oceanicus*	0.06	−0.11	–	–
*Teleogryllus occipitalis*	0.05	−0.12	–	–
*Teleogryllus occipitalis*	0.05	−0.12	–	–
*Teleogryllus occipitalis	0.05	−0.12		
*Tarbinskiellus portentosus*	0.06	−0.11	0.07	−0.06
*Tarbinskiellus portentosus*	0.05	−0.12	0.08	−0.12
*Tarbinskiellus portentosus	0.05	−0.12		
*Tarbinskiellus* sp.	0.06	−0.11	–	–
*Velarifictorus hemelytrus*	0.03	−0.15	0.15	−0.41
*Sclerogryllus punctatus*	0.05	−0.17	0.07	−0.18
*Turanogryllus eous*	0.07	−0.16	0.10	−0.24
subfamily Gryllinae	0.05^b^	−0.14^b^		

### Protein-coding genes and codon usage

Nine of the 13 PCGs were situated on the J-strand (represented clockwise in Figs. 1–3) and four on the N-strand (represented counter clockwise in Figs. 1–3). All PCGs started with ATN codons with the exception of *cox1* (TCG in *X. lamottei*) and *nd1* (TTG in all three eneopterine crickets). Nine genes shared same start codons among three species, while it is different in *atp8*, *cox1*, *nd3*, and *nd6* genes (Table 1). In the three species, the most common start codon was ATG, which was found in six PCGs, followed by ATT (four in *X. lamottei* and *N. vittatus*, three in *X. maniema*). In terms of stop codons, most PCGs terminated with TAA (seven in *X. maniema* and *N. vittatus*, six in *X. lamottei*) but TAG stop codon was observed with genes *cytb* and *nd3* in *N. vittatus*, *nd1* and *nd2* in *X. lamottei*, and *nd1* in *X. maniema*. Moreover, five incomplete stop codons (T) were observed with genes *cox1*, *cox2*, *cox3*, *nd4*, and *nd5* in *X. lamottei* and *X. maniema*, and four were in *N. vittatus* with genes *cox3*, *nd1*, *nd4*, and *nd5*.

The number of each codon, the relative synonymous codon usage (RSCU) values and the amino acid compositions of PCGs in the three eneopterines are given in Fig. S1. The pattern of codon usage was consistent with the preference for AT-rich codons in three eneopterines. Synonymous codons ending with A or T were clearly preferred, and UUA (Leu) were the most frequently used codons, followed by UCA (Ser) with exception of UCU (Ser) in *X. lamottei*.

**Figure 5:**
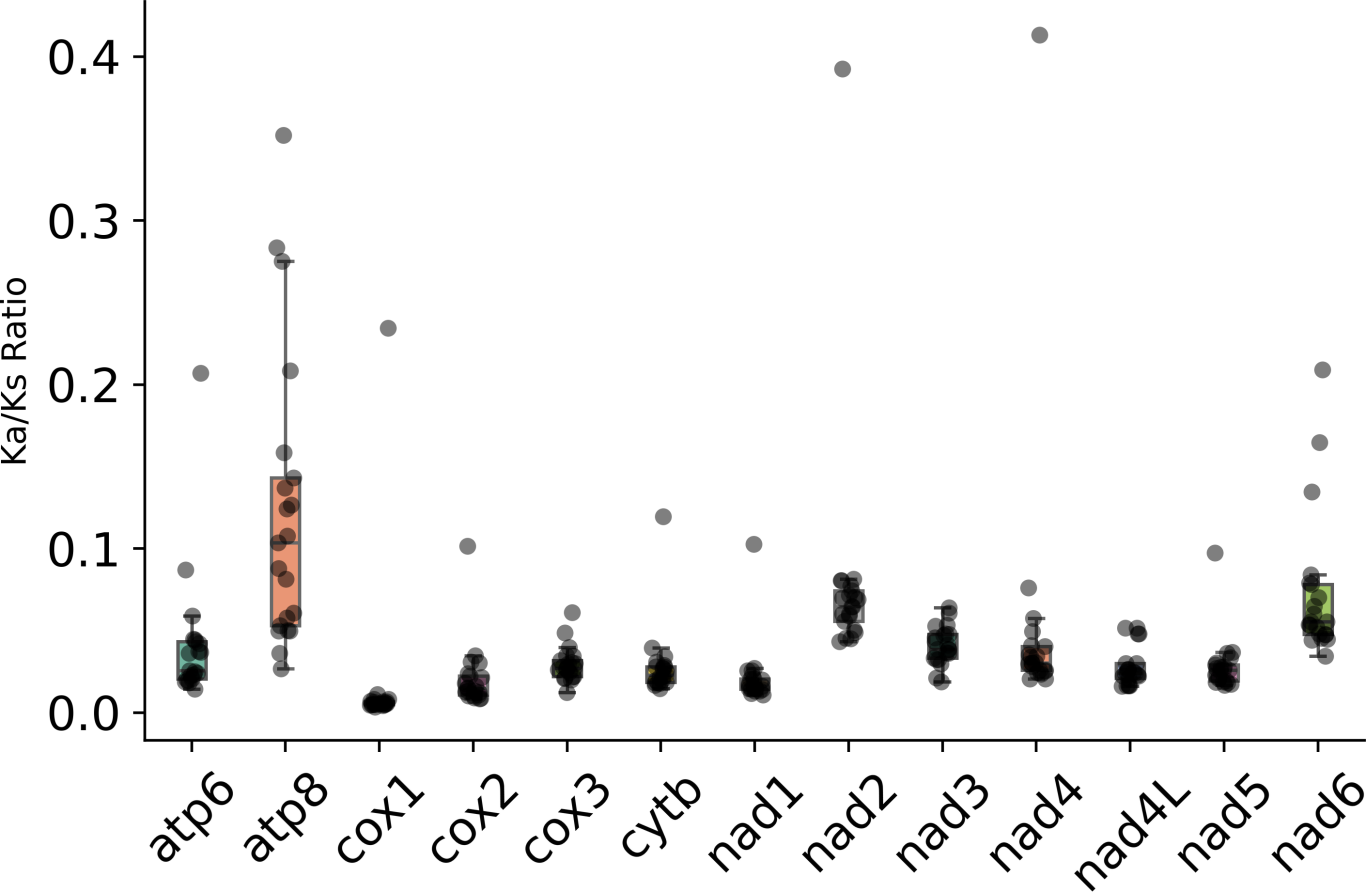
Ka/Ks ratio of PCGs in the subfamily Eneopterinae.

The Ka/Ks ratio of PCGs in the subfamily Eneopterinae is shown in Fig. 5. The ratios of Ka/Ks were all less than 1, indicating the existence of purifying selection in these species. Among the 13 PCGs, *atp*8 has experienced the strongest purifying pressure, followed by *nd*6 and *nd*2. However, two cytochrome c oxidase subunit genes (*cox1* and *cox2*) and *nd1* have experienced relatively weak purifying pressure. Moreover, *cytb* and *nd4l* probably have experienced the same level of purifying pressure.

### Transfer and ribosomal RNA genes

The tRNA genes ranged in size from 62 bp (*trnC* and *trnR*, both in *X. lamottei* and *X. maniema*, *trnH* in *N. vittatus*) to 72 bp (*trnK* in *X. maniema*). Apart from trnS1 (*AGN*) that lacked a stable dihydrouridine arm (DHU) (Fig. S2), all tRNA genes could be folded into typical clover-leaf secondary structures. The 22 tRNAs each shared the same anticodon among the three species.

Two rRNA genes, *rrnL* and *rrnS*, were encoded by the minority strand and separated by *trnV*. The *rrnL* ranged in size from 1,274 to 1,288 bp and located between *trnL* and *trnV* in the three eneopteries, while *rrnS* ranged in size from 748 to 772 bp and occurred between *trnV* and CR (Table 1 and Figs. 1–3). Each rRNA gene had a similar A+T content among these mitogenomes (Table 2).

### A+T-rich region

The CR was characterized by a high A+T content with 80.1% in *X. lamottei*, 78.3% in *X. maniema*, and 82.1% in *N. vittatus*, respectively (Table 2). It ranged in size from 414 bp in *N. vittatus* to 722 bp in *X. maniema*. The tandem repeats were detected in *N. Vittatus* (143 bp) but not in *X. lamottei* or *X. maniema*.

### Phylogenetic analyses

**Figure 6:**
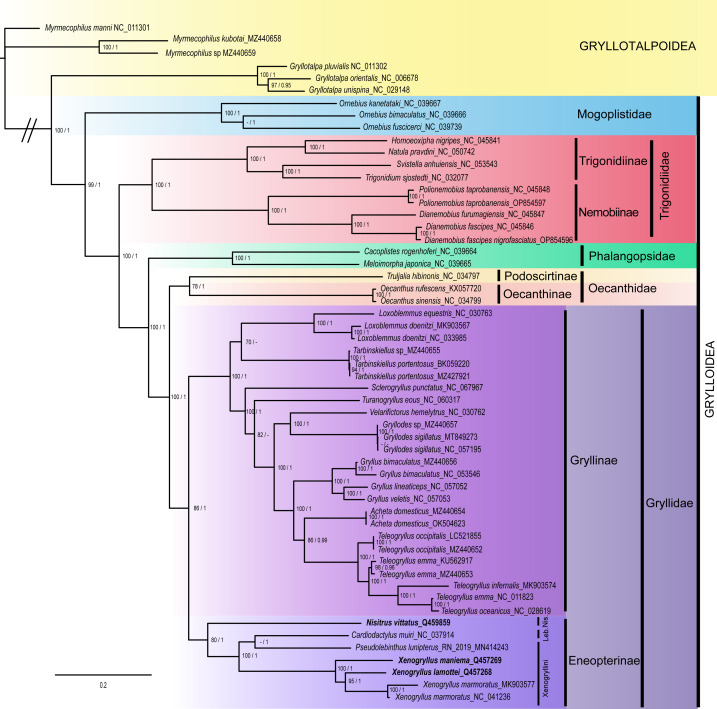
Phylogeny of the superfamily Grylloidea inferred from maximum likelihood (ML) and Bayesian inference (BI). Values on nodes indicate branch support; the first values correspond to non-parametric bootstrap values (BS) from IQ-TREE analyses, whereas the second value corresponds to BI posterior probabilities (PP). Signs “-” indicate topological incongruences between analyses. Species represented in bold correspond to the newly generated mitogenomes. The scale bar indicates the number of substitutions per site.

The topology of BI tree is similar to that of ML tree (Fig. 6; see also Appendix Fig. S3 for original outputs of both BI and ML analyses). The superfamily Grylloidea is recovered monophyletic with a high support (BS = 99%, PP = 1). The five main families of Grylloidea are also recovered monophyletic with high support: Mogoplistidae, represented by three *Ornebius* species is strongly supported (BS = 100%, PP = 1); it is placed as the sister clade of a group including the four other families, which is highly supported (BS = 100%, PP = 1). Trigonidiidae is found monophyletic with a high support (BS = 100%, PP = 1), and is sister to a clade grouping Phalangopsidae and a clade grouping Oecanthidae and Gryllidae, each family being monophyletic with a high support (BS = 100%, PP = 1 for Phalangopsidae; BS = 78%, PP = 1 for Oecanthidae; BS = 86%, PP = 1 for Gryllidae. In both ML and BI, both the subfamilies Eneopterinae and Gryllinae were recovered as monophyletic with good support (Eneopterinae: BS = 80%, PP = 1; Gryllinae: BS = 100%, PP = 1).

Within the Eneopterinae subfamily, the tribe Xenogryllini is not recovered as monophyletic, the species *Pseudolebinthus lunipterus* grouping with *Cardiodactylus muiri* (tribe Lebinthini). The genus *Xenogryllus* is strongly supported as a monophyletic group in both analyses (BS = 100%, PP = 1).

## Discussion

In this study, we generated three mitogenomes for the cricket subfamily Eneopterinae, which allows comparisons with existing mitogenomes from other cricket subfamilies.

The gene order and orientation within the mitogenome of *X. lamottei*, *X. maniema*, and *N. vittatus* are identical with those described for other Gryllidae crickets. The length of the whole mitogenomes and the length range of tRNAs in these three species are also similar to other subfamilies. The new mitogenomes are weakly AT-skewed and strongly GC-skewed. The nucleotide compositional AT-skew of whole mitogenome, PCGs, *rrnL*, and tRNAs are slightly stronger in the subfamily Eneopterinae than that in Gryllinae, but it is yet too early to test the significance of this trend, as there are too few data available at the scale of the family. Therefore, more mitogenomes of other subfamilies are necessary to test whether this is consistent among the subfamilies. Overall, the results of the genome skimming method are fully satisfactory compared to other NGS methods in order to recover mitogenomic data from museum specimens, as suggested by previous studies (Nattier & Salazar, 2019; Salazar & Nattier, 2020; Zeng et al., 2018); despite lower coverage and DNA degradation, the quality and length distribution of the sequences obtained from museum specimens are sufficient to pass quality tests and are generally comparable with data obtained from fresher material.

Interestingly, the evolutionary rates of *apt8* and *nd6* are higher than that of any other mitochondrial genes in eneopterine crickets according to the Ka/Ks ratios. This is consistent with previous results about another eneopterine species from the tribe Lebinthini (Dong et al., 2017), and suggests that these higher rates are common to all the members of the subfamily Eneopterinae. It is not a surprise that the cytochrome c oxidase subunit genes have experienced relatively weak purifying pressure according to their Ka/Ks ratios, and it is probably correlated with the fact that *cox1* is regularly used as a barcoding marker, for it shows less intraspecific than interspecific variation.

The phylogenetic analyses recover the main topologies found in previous studies at the scale of the familial and subfamilial relationship all the families and subfamilies currently recognized in crickets and that were tested by our taxonomic sampling were recovered with high support. Our study overall attests that the mitogenomic data strongly support the phylogenetic relationships at multiple time scales: all the families within the superfamily Grylloidea and their relationships are confirmed despite the low taxonomic sampling in this study. The deeper nodes in the cricket phylogeny that are recovered by our study are consistent with the conclusions of recent molecular phylogenetic studies (Chintauan-Marquier et al., 2016; de Campos et al., 2022; Ma et al., 2019; Ma, Zhang & Li, 2019; Song et al., 2015; Song et al., 2020). Our results also attest that the mitogenomic information is valuable to resolve the phylogenetic relationships within the family Gryllidae, from the monophyly of the subfamilies to their relationships. In the subfamily Eneopterinae, our results also show that mitogenomic data perform well to recover generic and species-level relationships, while they are less accurate to recover tribal relationships previously supported by recent papers on the phylogeny of this subfamily (Dong et al., 2018; Jaiswara et al., 2019; Vicente et al., 2017). The relationships within the subfamily Eneopterinae are not similar in our study given the position of species *C. muiri*, found as the sister group of *P. lunipterus*. These differences could be explained by sampling effects, and to the fact that the diverse Lebinthini tribe is only represented by one *Cardiodactylus* species (*C. muiri*) in our study.

Finally, it is important to recall that two of the newly generated mitogenomes correspond to dry-pinned historical specimens from natural history collections respectively 80 and 68 years old (age at date of extraction). The genome skimming method used for sequencing performed as well as for the recent specimen used for the third cricket species, confirming how promising this approach is to perform large-scale comparative studies of mitogenomes to promote resources existing in natural history collections (Nattier & Salazar, 2019).

## Conclusion

We show that the genome skimming method used by NGS allowed to efficiently obtain the whole mitogenome from dry-pinned historical specimens. Genome skimming is a promising method to break the limitation by the availability of sequence material for species known from only a few old specimens and/or from regions difficult to access and take full advantage of using the natural history collection specimens. Additionally, the new mitogenomes described in the present study, combined with the existing datasets, arises as an invaluable resource for future comparative evolutionary genomic studies in insects.

## Supplemental Information

10.7717/peerj.17734/supp-1 Supplemental Information 1 Relative synonymous codon usage (RSCU) in the mitogenomes of *N. vittatus*, *X. lamottei* and *X. maniema* The stop codon is not given. The different color areas indicate the proportions of codons in each amino acid.

10.7717/peerj.17734/supp-2 Supplemental Information 2 Secondary structure of tRNAs of *N. vittatus*

10.7717/peerj.17734/supp-3 Supplemental Information 3 Secondary structure of tRNAs of *X. lamottei.*

10.7717/peerj.17734/supp-4 Supplemental Information 4 Secondary structure of tRNAs of *X. maniema.*

10.7717/peerj.17734/supp-5 Supplemental Information 5 Original outputs of BI and ML trees

10.7717/peerj.17734/supp-6 Supplemental Information 6 The material information of sampling taxa in this study The abbreviation of repository indicates: Muséum national d’Histoire naturelle, Paris (MNHN) and Musée Royal de l’Afrique Centrale, Tervuren, Belgium (MRAC).

10.7717/peerj.17734/supp-7 Supplemental Information 7 Taxa used for phylogenetic analyses in this study Genbank accession number with * indicates that it obtained in this study.

10.7717/peerj.17734/supp-8 Supplemental Information 8 Raw mitogenomic sequences of *Nisitrus vittatus* The sequence was deposited in GenBank under accession number OQ459859 (molecular code: N37).

10.7717/peerj.17734/supp-9 Supplemental Information 9 Raw mitogenomic sequences of *Xenogryllus lamottei* The sequence was deposited in GenBank under accession number OQ457268 (molecular code: X24).

10.7717/peerj.17734/supp-10 Supplemental Information 10 Raw mitogenomic sequences of *Xenogryllus maniema* The sequence was deposited in GenBank under accession number OQ457269 (molecular code: X36).

## Additional Information and Declarations

### Field Study Permissions

The following information was supplied relating to field study approvals (i.e., approving body and any reference numbers): The collection of *N. vittatus* material in Kuala Belalong Field Studies Centre, Brunei Darussalam was granted by the Institute for Biodiversity and Environmental Research, Universiti Brunei Darussalam (UBD/AVC-RI/1.21.1 [a]) and the export permit was issued by the Research Innovation Centre (BioRIC), Ministry of Primary Resources and Tourism, Brunei Darussalam (BioRIC/HoB/TAD/51-73).

### Data Availability

The following information was supplied regarding data availability: The sequences are available at NCBI: OQ457268, OQ457269 and and OQ459859 and the raw mitogenomic sequences are provided in the Supplementary Files.
