# Supplementary figures and images for: Museomics allows comparative analyses of mitochondrial genomes in the family Gryllidae (Insecta, Orthoptera) and confirms its phylogenetic relationships
Source: PeerJ. 2024 Aug 8;12:e17734. doi: 10.7717/peerj.17734 (PMC11317039; doi:10.7717/peerj.17734)

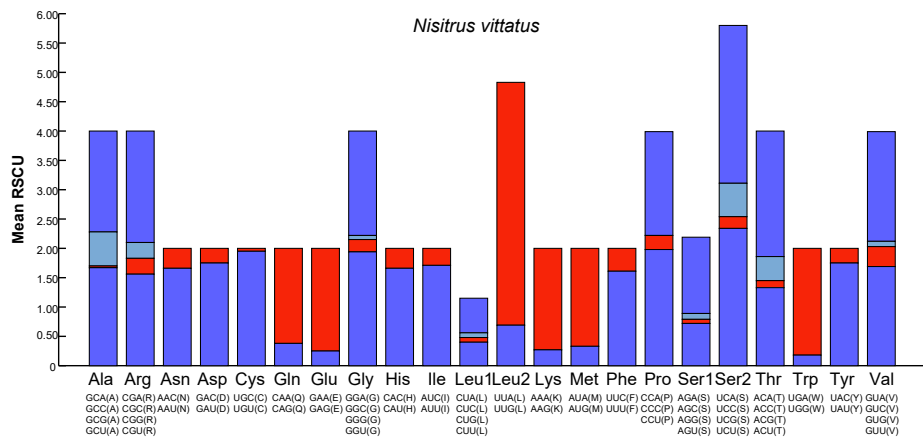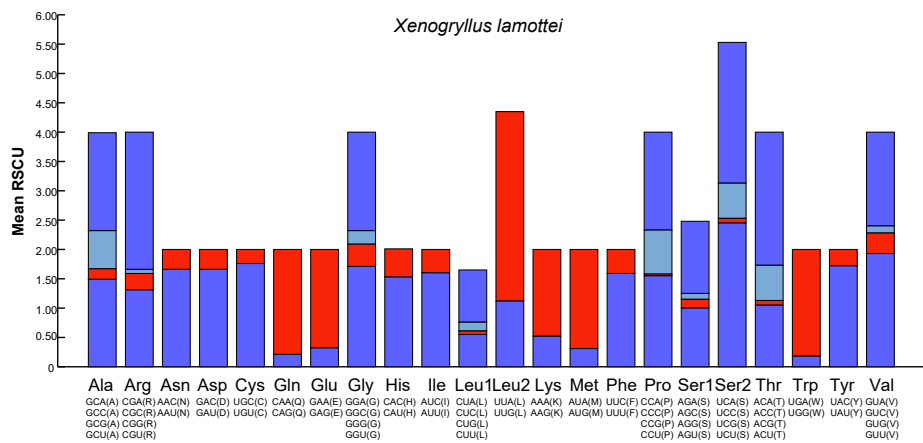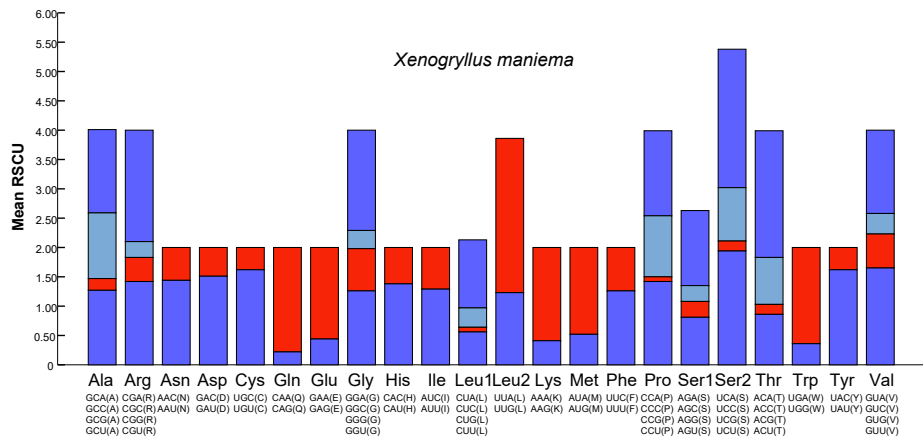

Supplement: Supplemental Information 1 — The stop codon is not given. The different color areas indicate the proportions of codons in each amino acid. [file peerj-12-17734-s001.pdf]

a

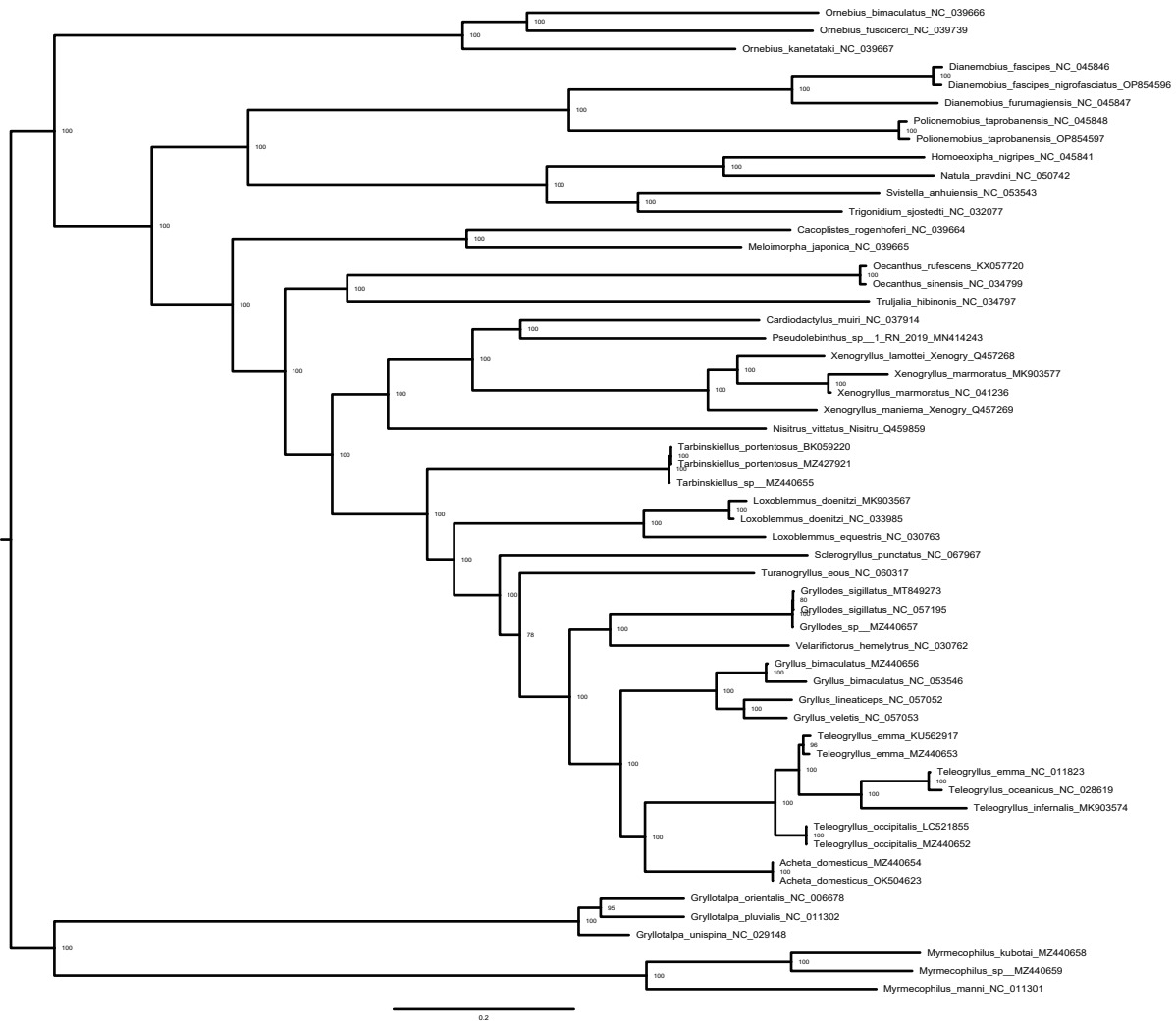

b

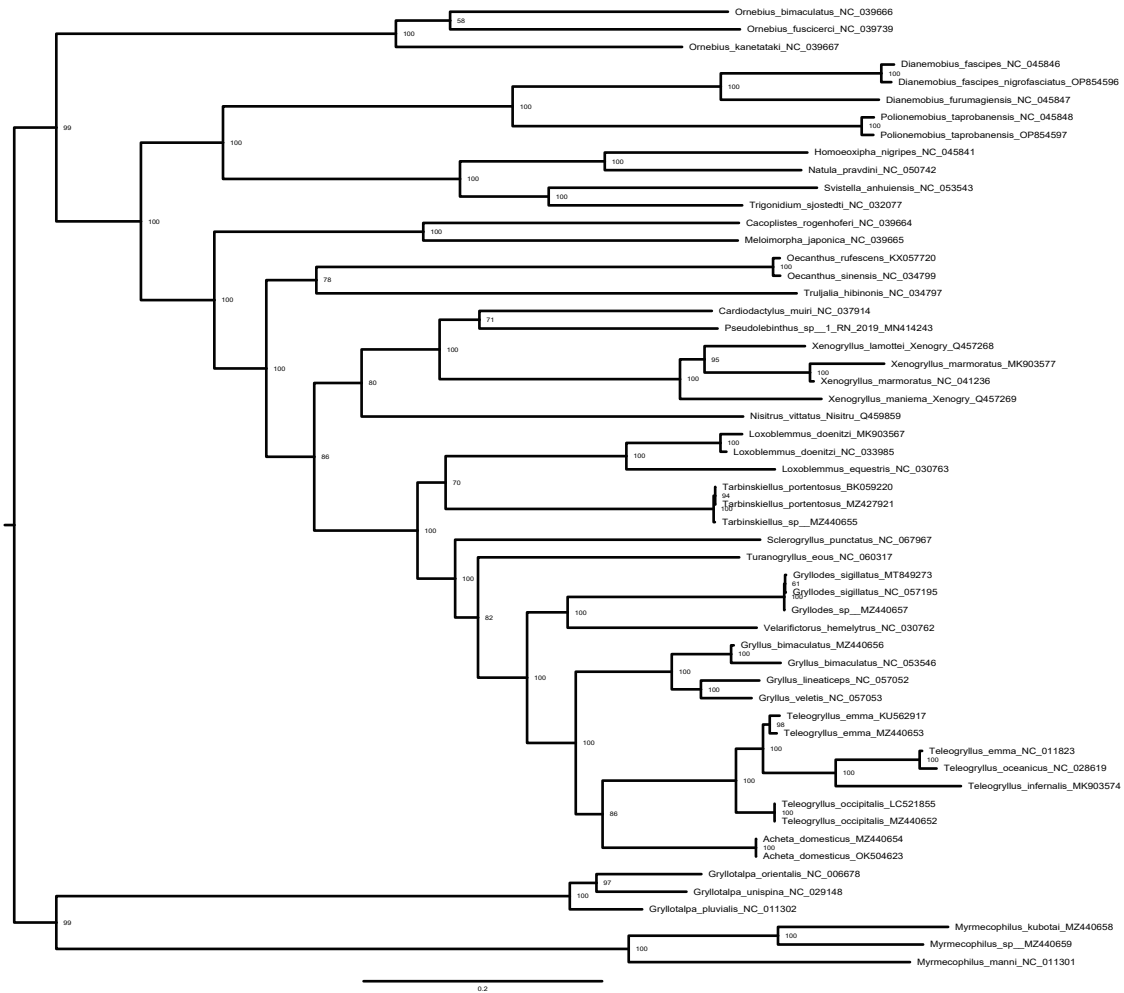

Supplement: Supplemental Information 5 [file peerj-12-17734-s005.pdf]
