# Supplementary material for: Museomics allows comparative analyses of mitochondrial genomes in the family Gryllidae (Insecta, Orthoptera) and confirms its phylogenetic relationships
Source: PeerJ. 2024 Aug 8;12:e17734. doi: 10.7717/peerj.17734 (PMC11317039; doi:10.7717/peerj.17734)
Supplement: Supplemental Information 2 [file peerj-12-17734-s002.pdf]

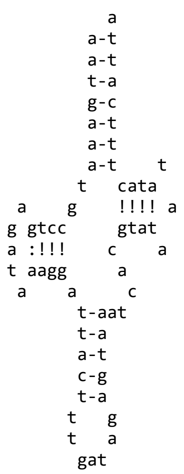
tRNA-Ile(gat)

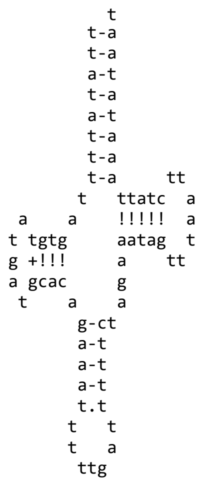
tRNA-Gln(ttg)

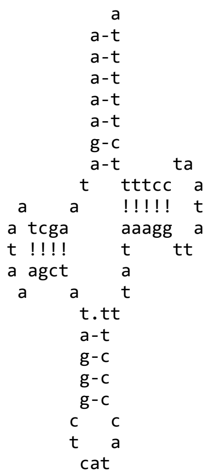
tRNA-Met(cat)

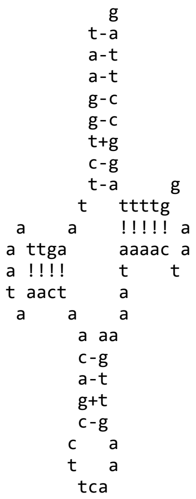
tRNA-Trp(tca)

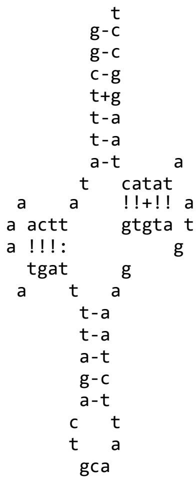
tRNA-Cys(gca)

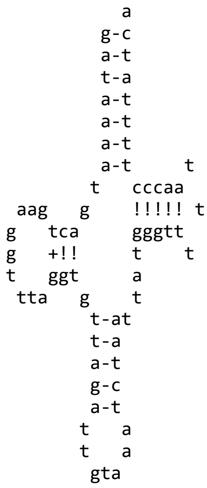
tRNA-Tyr(gta)

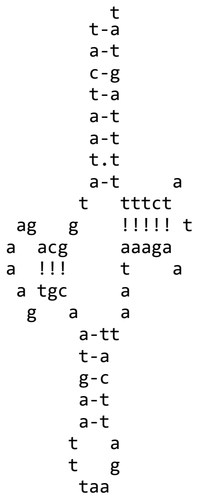
tRNA-Leu(taa)

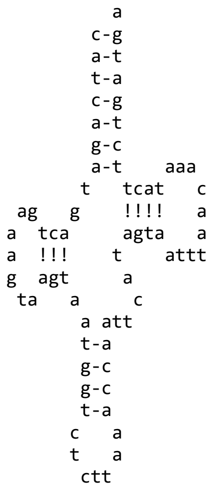
tRNA-Lys(ctt)

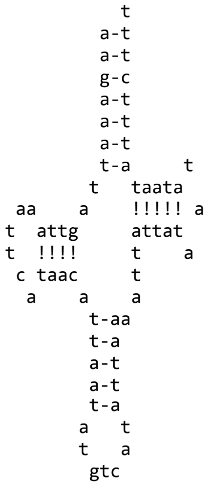
tRNA-Asp(gtc)

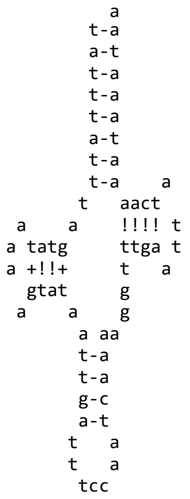
tRNA-Gly(tcc)

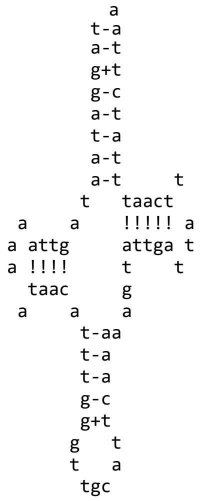
tRNA-Ala(tgc)

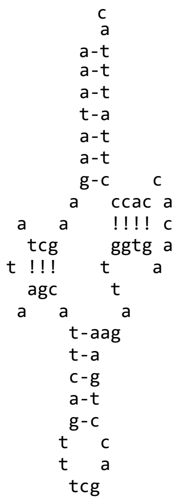
tRNA-Arg(tcg)

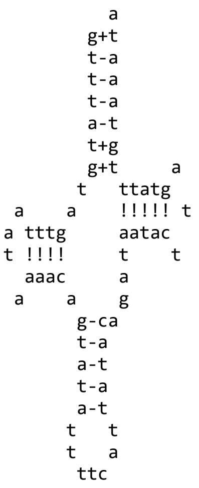
tRNA-Glu(ttc)

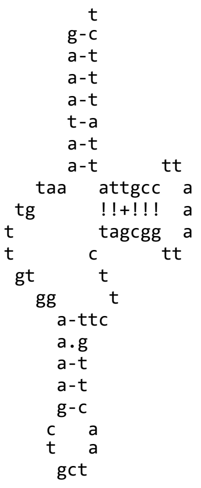
tRNA-Ser(gct)

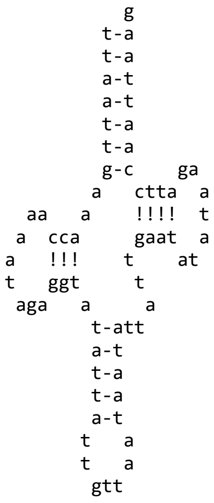
tRNA-Asn(gtt)

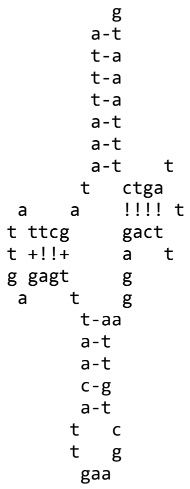
tRNA-Phe(gaa)

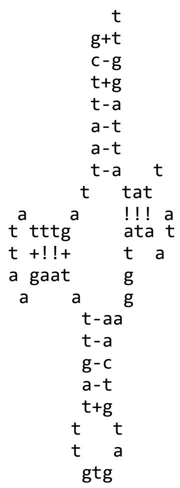
tRNA-His(gtg)

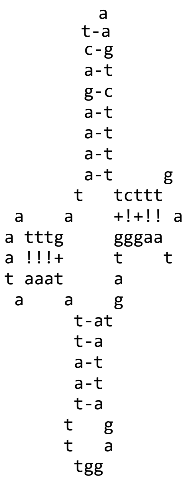
tRNA-Pro(tgg)

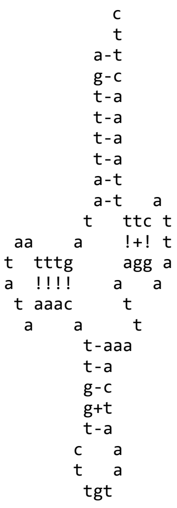
tRNA-Thr(tgt)

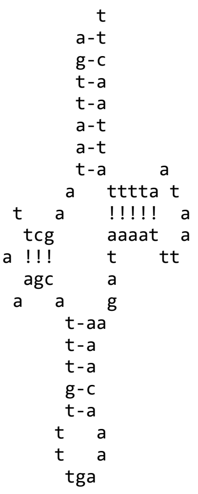
tRNA-Ser(tga)

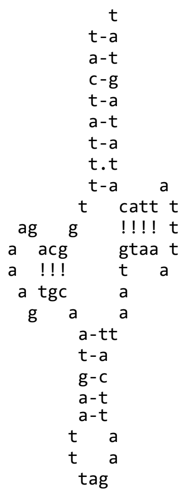
tRNA-Leu(tag)

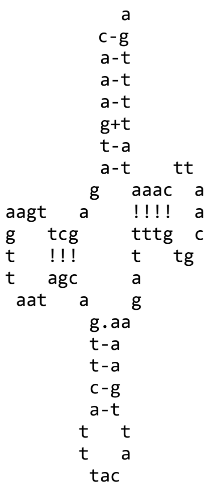
tRNA-Val(tac)
