# Supplementary material for: Museomics allows comparative analyses of mitochondrial genomes in the family Gryllidae (Insecta, Orthoptera) and confirms its phylogenetic relationships
Source: PeerJ. 2024 Aug 8;12:e17734. doi: 10.7717/peerj.17734 (PMC11317039; doi:10.7717/peerj.17734)
Supplement: Supplemental Information 4 [file peerj-12-17734-s004.pdf]

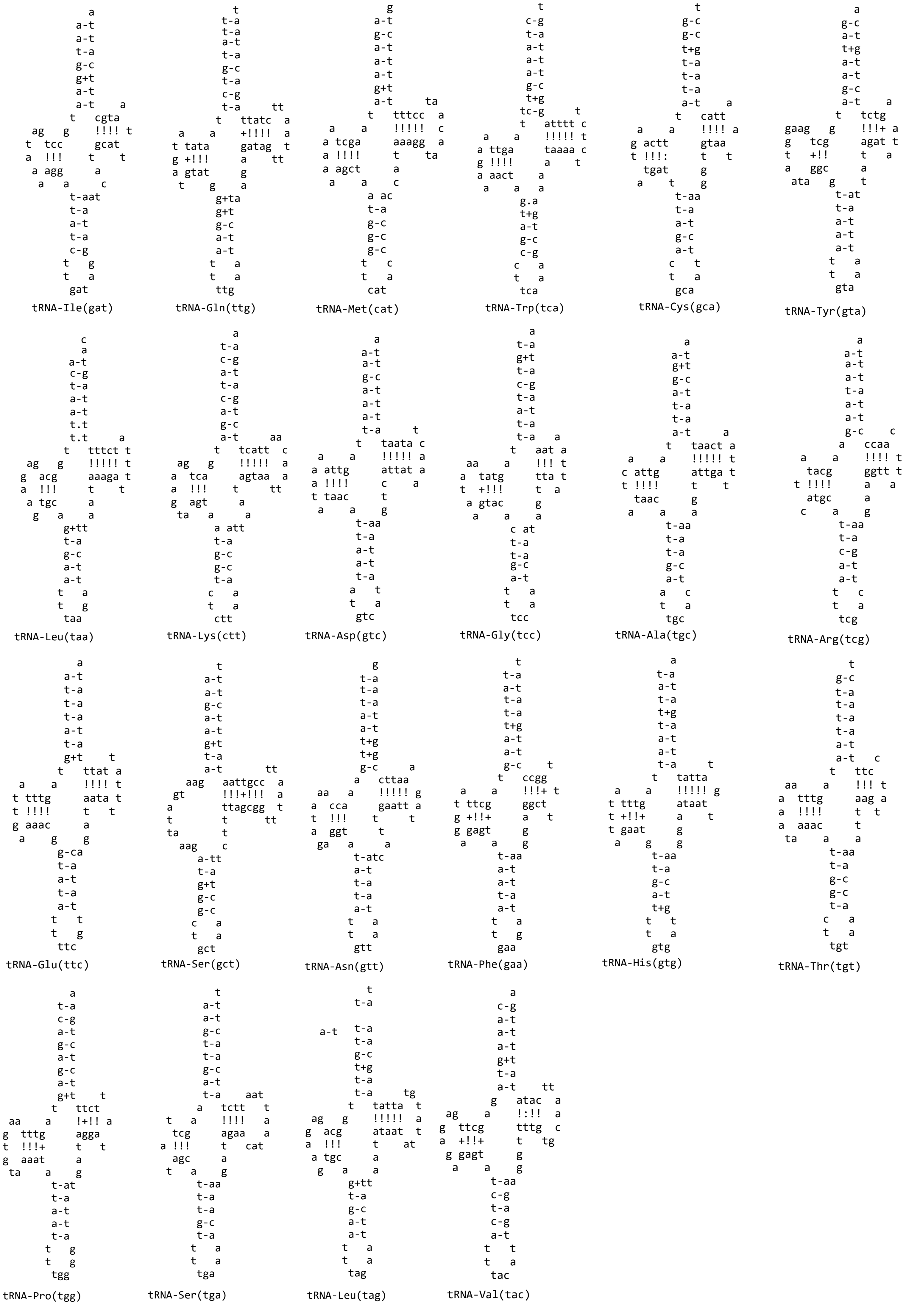
tRNA-Ile(gat)
tRNA-Gln(ttg)
tRNA-Met(cat)
tRNA-Trp(tca)
tRNA-Cys(gca)
tRNA-Tyr(gta)
tRNA-Leu(taa)
tRNA-Lys(ctt)
tRNA-Asp(gtc)
tRNA-Gly(tcc)
tRNA-Ala(tgc)
tRNA-Arg(tcg)
tRNA-Glu(ttc)
tRNA-Ser(gct)
tRNA-Asn(gtt)
tRNA-Phe(gaa)
tRNA-His(gtg)
tRNA-Thr(tgt)
tRNA-Pro(tgg)
tRNA-Ser(tga)
tRNA-Leu(tag)
tRNA-Val(tac)
