# Supplementary material for: Museomics allows comparative analyses of mitochondrial genomes in the family Gryllidae (Insecta, Orthoptera) and confirms its phylogenetic relationships
Source: PeerJ. 2024 Aug 8;12:e17734. doi: 10.7717/peerj.17734 (PMC11317039; doi:10.7717/peerj.17734)
Supplement: Supplemental Information 6 — The abbreviation of repository indicates: Muséum national d’Histoire naturelle, Paris (MNHN) and Musée Royal de l’Afrique Centrale, Tervuren, Belgium (MRAC). [file peerj-12-17734-s006.docx]

Table S1. The material information of sampling taxa in this study.

|  | *Nisitrus vitattus* | *Xenogryllus lamottei* | *Xenogryllus maniema* |
| --- | --- | --- | --- |
| Taxon status (Tribe) | Nisitrini | Xenogryllini | Xenogryllini |
| Type status |  | holotype | paratype |
| Collection date | 8-I-2017 | IX-1951 | IX-1939 |
| Locality | Kuala Belalong, Brunei Darussalam | Simandou Mount, Guinea | Lokandu, Island, Biawa, Democratic Republic of the Congo |
| Repository | MNHN | MNHN | MRAC |
| Specimen voucher | MNHN-EO-ENSIF11069 | MNHN-EO-ENSIF10685 | MRAC |
| Molecular code | N37 | X24 | X36 |
| Acccession number | OQ459859 | OQ457268 | OQ457269 |

The abbreviation of repository indicates: Muséum national d’Histoire naturelle, Paris (MNHN) and Musée Royal de l'Afrique Centrale, Tervuren, Belgium (MRAC).
