# Supplementary material for: Museomics allows comparative analyses of mitochondrial genomes in the family Gryllidae (Insecta, Orthoptera) and confirms its phylogenetic relationships
Source: PeerJ. 2024 Aug 8;12:e17734. doi: 10.7717/peerj.17734 (PMC11317039; doi:10.7717/peerj.17734)
Supplement: Supplemental Information 7 — Genbank accession number with * indicates that it obtained in this study. [file peerj-12-17734-s007.docx]

**Table S2.** Taxa used for phylogenetic analyses in this study.

| Superfamily | Family | Subfamily | Tribe | Species | GenBank accession number |
| --- | --- | --- | --- | --- | --- |
| Grylloidea | Gryllidae | Eneopterinae | Lebinthini | *Cardiodactylus muiri* Otte, 2007 | NC_037914 |
| Grylloidea | Gryllidae | Eneopterinae | Nisitrini | *Nisitrus vittatus* (Haan, 1844) | OQ459859* |
| Grylloidea | Gryllidae | Eneopterinae | Xenogryllini | *Pseudolebinthus lunipterus* Salazar et al, 2020 | MN414243 |
| Grylloidea | Gryllidae | Eneopterinae | Xenogryllini | *Xenogryllus lamottei* Robillard, 2019 | OQ457268* |
| Grylloidea | Gryllidae | Eneopterinae | Xenogryllini | *Xenogryllus maniema* Robillard & Jaiswara, 2019 | OQ457269* |
| Grylloidea | Gryllidae | Eneopterinae | Xenogryllini | *Xenogryllus marmoratus* (Haan, 1844) | NC_041236 |
| Grylloidea | Gryllidae | Eneopterinae | Xenogryllini | *Xenogryllus marmoratus* (Haan, 1845) | MK903577 |
| Grylloidea | Gryllidae | Gryllinae | Gryllini | *Acheta domesticus* (Linnaeus, 1758) | OK504623 |
| Grylloidea | Gryllidae | Gryllinae | Gryllini | *Acheta domesticus* (Linnaeus, 1759) | MZ440654 |
| Grylloidea | Gryllidae | Gryllinae | Gryllini | *Gryllodes sigillatus* (Walker, 1869) | NC_057195 |
| Grylloidea | Gryllidae | Gryllinae | Gryllini | *Gryllodes sigillatus* (Walker, 1869) | MT849273 |
| Grylloidea | Gryllidae | Gryllinae | Gryllini | *Gryllodes sp.* | MZ440657 |
| Grylloidea | Gryllidae | Gryllinae | Gryllini | *Gryllus bimaculatus* De Geer, 1773 | NC_053546 |
| Grylloidea | Gryllidae | Gryllinae | Gryllini | *Gryllus bimaculatus* De Geer, 1773 | MZ440656 |
| Grylloidea | Gryllidae | Gryllinae | Gryllini | *Gryllus lineaticeps* Stål, 1861 | NC_057052 |
| Grylloidea | Gryllidae | Gryllinae | Gryllini | *Gryllus veletis* (Alexander & Bigelow, 1960) | NC_057053 |
| Grylloidea | Gryllidae | Gryllinae | Gryllini | *Loxoblemmus doenitzi* Stein, 1881 | NC_033985 |
| Grylloidea | Gryllidae | Gryllinae | Gryllini | *Loxoblemmus doenitzi* Stein, 1881 | MK903567 |
| Grylloidea | Gryllidae | Gryllinae | Gryllini | *Loxoblemmus equestris* Saussure, 1877 | NC_030763 |
| Grylloidea | Gryllidae | Gryllinae | Gryllini | *Teleogryllus emma* (Ohmachi & Matsuura, 1951) | KU562917 |
| Grylloidea | Gryllidae | Gryllinae | Gryllini | *Teleogryllus emma* (Ohmachi & Matsuura, 1952) | MZ440653 |
| Grylloidea | Gryllidae | Gryllinae | Gryllini | *Teleogryllus emma* (Ohmachi & Matsuura, 1952) | NC_011823 |
| Grylloidea | Gryllidae | Gryllinae | Gryllini | *Teleogryllus infernalis* | MK903574 |
| Grylloidea | Gryllidae | Gryllinae | Gryllini | *Teleogryllus oceanicus* (Le Guillou, 1841) | NC_028619 |
| Grylloidea | Gryllidae | Gryllinae | Gryllini | *Teleogryllus occipitalis* (Serville, 1838) | MZ440652 |
| Grylloidea | Gryllidae | Gryllinae | Gryllini | *Teleogryllus occipitalis* (Serville, 1839) | LC521855 |
| Grylloidea | Gryllidae | Gryllinae | Gryllini | *Tarbinskiellus portentosus* (Lichtenstein, 1796) | BK059220 |
| Grylloidea | Gryllidae | Gryllinae | Gryllini | *Tarbinskiellus portentosus* (Lichtenstein, 1796) | MZ427921 |
| Grylloidea | Gryllidae | Gryllinae | Gryllini | *Tarbinskiellus sp* | MZ440655 |
| Grylloidea | Gryllidae | Gryllinae | Modicogryllini | *Velarifictorus hemelytrus* (Saussure, 1877) | NC_030762 |
| Grylloidea | Gryllidae | Gryllinae | Sclerogryllini | *Sclerogryllus punctatus* (Brunner von Wattenwyl, 1893) | NC_067967 |
| Grylloidea | Gryllidae | Gryllinae | Turanogryllini | *Turanogryllus eous* Bey-Bienko, 1956 | NC_060317 |
| Grylloidea | Mogoplistidae | Mogoplistinae | Arachnocephalini | *Ornebius kanetataki* (Matsumura, 1904) | NC_039667 |
| Grylloidea | Mogoplistidae | Mogoplistinae | Arachnocephalini | *Ornebius bimaculatus* (Shiraki, 1930) | NC_039666 |
| Grylloidea | Mogoplistidae | Mogoplistinae | Arachnocephalini | *Ornebius fuscicercis* (Shiraki, 1930) | NC_039739 |
| Grylloidea | Oecanthidae | Oecanthinae | Oecanthini | *Oecanthus rufescens* Serville, 1838 | KX057720 |
| Grylloidea | Oecanthidae | Oecanthinae | Oecanthini | *Oecanthus sinensis* Walker, 1869 | NC_034799 |
| Grylloidea | Oecanthidae | Podoscirtinae | Podoscirtini | *Truljalia hibinonis* (Matsumura, 1917) | NC_034797 |
| Grylloidea | Phalangopsidae | Cachoplistinae | Cachoplistini | *Cacoplistes rogenhoferi* Saussure, 1877 | NC_039664 |
| Grylloidea | Phalangopsidae | Cachoplistinae | Homoeogryllini | *Meloimorpha japonica* (Haan, 1844) | NC_039665 |
| Grylloidea | Trigonidiidae | Trigonidiinae | Trigonidiini | *Homoeoxipha nigripes* Xia & Liu, 1993 | NC_045841 |
| Grylloidea | Trigonidiidae | Trigonidiinae | Trigonidiini | *Trigonidium sjostedti* (Chopard, 1925) | NC_032077 |
| Grylloidea | Trigonidiidae | Trigonidiinae | Trigonidiini | *Svistella anhuiensis* He, Li & Liu, 2009 | NC_053543 |
| Grylloidea | Trigonidiidae | Trigonidiinae | Trigonidiini | *Natula pravdini* (Gorochov, 1985) | NC_050742 |
| Grylloidea | Trigonidiidae | Nemobiinae | Pteronemobiini | *Dianemobius fascipes* (Walker, 1869) | NC_045846 |
| Grylloidea | Trigonidiidae | Nemobiinae | Pteronemobiini | *Dianemobius fascipes nigrofasciatus* (Matsumura, 1904) | OP854596 |
| Grylloidea | Trigonidiidae | Nemobiinae | Pteronemobiini | *Dianemobius furumagiensis* (Ohmachi & Furukawa, 1929) | NC_045847 |
| Grylloidea | Trigonidiidae | Nemobiinae | Pteronemobiini | *Polionemobius taprobanensis* Gorochov, 1986 | NC_045848 |
| Grylloidea | Trigonidiidae | Nemobiinae | Pteronemobiini | *Polionemobius taprobanensis* (Walker, 1869) | OP854597 |
| Gryllotalpoidea | Gryllotalpidae | Gryllotalpinae | Gryllotalpini | *Gryllotalpa orientalis* Burmeister, 1838 | NC_006678 |
| Gryllotalpoidea | Gryllotalpidae | Gryllotalpinae | Gryllotalpini | *Gryllotalpa pluvialis* (Mjöberg, 1913) | NC_011302 |
| Gryllotalpoidea | Gryllotalpidae | Gryllotalpinae | Gryllotalpini | *Gryllotalpa unispina* Saussure, 1874 | NC_029148 |
| Gryllotalpoidea | Myrmecophilidae | Myrmecophilinae | Myrmecophilini | *Myrmecophilus manni* Schimmer, 1911 | NC_011301 |
| Gryllotalpoidea | Myrmecophilidae | Myrmecophilinae | Myrmecophilini | *Myrmecophilus kubotai* Maruyama, 2004 | MZ440658 |
| Gryllotalpoidea | Myrmecophilidae | Myrmecophilinae | Myrmecophilini | *Myrmecophilus sp* | MZ440659 |

Genbank accession number with * indicates that it obtained in this study.
